# Postnatal Dexamethasone Therapy Impairs Brown Adipose Tissue Thermogenesis and Autophagy Flux in Neonatal Rat Pups

**DOI:** 10.7150/thno.70752

**Published:** 2022-07-25

**Authors:** Yu-Shan Chang, Shun-Yun Hou, Shang-Shiuan Yu, Shin-Yu Tsai, Ying-Yi Chen, Li-Jin Hsu, Pei-Jane Tsai, Hui-Kuan Lin, Chyi-Her Lin, Yau-Sheng Tsai

**Affiliations:** 1 Institute of Clinical Medicine, College of Medicine, National Cheng Kung University, Tainan, Taiwan, ROC; 2 Department of Pediatrics, National Cheng Kung University Hospital, College of Medicine, National Cheng Kung University, Tainan, Taiwan, ROC; 3 Department of Medical Laboratory Science and Biotechnology, College of Medicine, National Cheng Kung University, Tainan, Taiwan, ROC; 4 Department of Cancer Biology, Wake Forest Baptist Medical Center, Wake Forest University, Winston Salem, NC, 27101, USA; 5 Department of Pediatrics, E-Da Hospital, Post-baccalaureate Program, College of Medicine, I-Shou University, Kaohsiung, Taiwan, ROC; 6 Center for Clinical Medicine Research, National Cheng Kung University Hospital, College of Medicine, National Cheng Kung University, Tainan, Taiwan, ROC

**Keywords:** glucocorticoid, preterm infants, brown adipose tissue, mitochondria, autophagy

## Abstract

**Rationale:** Very preterm infants may require dexamethasone (Dex) for facilitating extubation or treating bronchopulmonary dysplasia. However, Dex may result in disturbance of metabolisms. This study was to investigate the effects of postnatal short course Dex exposure on brown adipose tissue (BAT) in neonatal rats.

**Method**: Neonatal rats received either three consecutive doses of daily Dex (0.2 mg/kg/day) or saline from postnatal P1 to P3. We investigated the effects of Dex on BAT including thermogenesis, mitochondrial dynamics and autophagy flux. We also compared diurnal temperature variation between preterm infants who received systemic corticosteroid and their treatment-naïve controls.

**Results**: Postnatal Dex treatment induced growth retardation, BAT whitening, UCP1 downregulation and cold intolerance in neonatal rats. BAT mitochondria were damaged, evident by loss of normal number, structure, and alignment of cristae. Mitochondrial fission-fusion balance was disrupted and skewed toward increased fusion, reflected by increased OPA1 and MFN2 and decreased DRP1, FIS1 and phosphorylated MFF protein levels. Autophagosome synthesis was increased but clearance was inhibited, indicated by accumulation of p62 protein after Dex treatment and no further increase of LC3-II after chloroquine co-treatment. While autophagy modulators, including chloroquine and rapamycin, did not improve UCP1 downregulation and BAT whitening, AMPK activators could partially rescue these damages. We also demonstrated that preterm infants had higher diurnal temperature variation during corticosteroid treatment.

**Conclusions**: Postnatal short course Dex impaired BAT mitochondrial function and autophagy flux in rat pups. AMPK activators had the potential to rescue the damage.

## Introduction

Dexamethasone (Dex) is used to treat or prevent bronchopulmonary dysplasia (BPD) of preterm infants, but is associated with cerebral palsy and adverse neurodevelopmental outcomes 1-3. The American Academy of Pediatrics revised policy statement on postnatal corticosteroid for BPD in 2010, and concluded that high-dose Dex cannot be recommended. Soll *et al.* reported that in the Vermont Oxford Network registry, 8% of very low-birth-weight infants still received postnatal corticosteroids and 23% of its use was in the highest risk group with birth weights of 501-750 g 4. Further evidence has suggested that the benefits of using Dex may outweigh the harms when the risk of BPD or cerebral palsy is high (> 50%) 5. The European consensus guidelines on the management of respiratory distress syndrome 2019 update still recommended a short course of low or very-low dose Dex to facilitate extubation in infants who remain on mechanical ventilation after 1-2 weeks of age 6-8. In the EPICE (Effective Perinatal Intensive Care in Europe) cohort, up to 49% of NICUs still use various regimens of postnatal corticosteroid, among which Dex accounts for nearly 40% of usage 9.

Hypothermia is a significant clinical problem in preterm infants and has been associated with increased mortality and major morbidities 10-12. The autonomic nerve system controls several effectors of temperature regulation including brown adipose tissue (BAT), vasomotor tone and sweat glands. However, in preterm infants, the vasomotor response to cold stress is lacking, especially during the first few days of life 13, 14. The efficiency of sweating as a thermoregulatory process is also poor 15. Therefore, BAT plays an important role in temperature regulation 16. Beyond thermogenesis, BAT is crucial in energy metabolism and has received much attention for treating obesity and diabetes 17-19. Uncoupling protein-1 (UCP1) short-circuits the electron transport chain, allowing mitochondrial membrane potential to be transduced to heat, making BAT a tissue capable of responding to energy expenditure and fuel metabolism in mammals.

Glucocorticoid (GC) inhibits the expression of UCP1 in adult rodents 20-22 and has various deleterious effects on mitochondria of different tissues 23-27. However, little is known about its effect on BAT mitochondria 28. Furthermore, whether and how GC affects autophagy and/or mitophagy in BAT remain unknown. A recent study by Deng *et al*. showed that inhibition of autophagy prevented BAT from Dex-induced whitening in adult mice 28. On the contrary, another study showed that impaired mitophagy led to BAT dysfunction and insulin resistance in adult mice 29. Since both models were examined in adult animals, the influence of GC on autophagy and/or mitophagy on neonatal BAT function remains unclear.

Clinical studies have shown that preterm infants receiving Dex therapy had reduced growth rate, decreased weight gain, and smaller head circumferences 30, 31. We speculate that Dex therapy may disrupt normal energy expenditure and BAT thermogenesis in neonates. Thus, this study was to investigate the effect of postnatal Dex exposure on BAT thermogenesis in neonatal rat pups. We hypothesized that exposure to Dex in neonates attenuates BAT thermogenesis through disturbance of mitochondrial function.

## Methods

### Animal experiment

**Figure 1:**
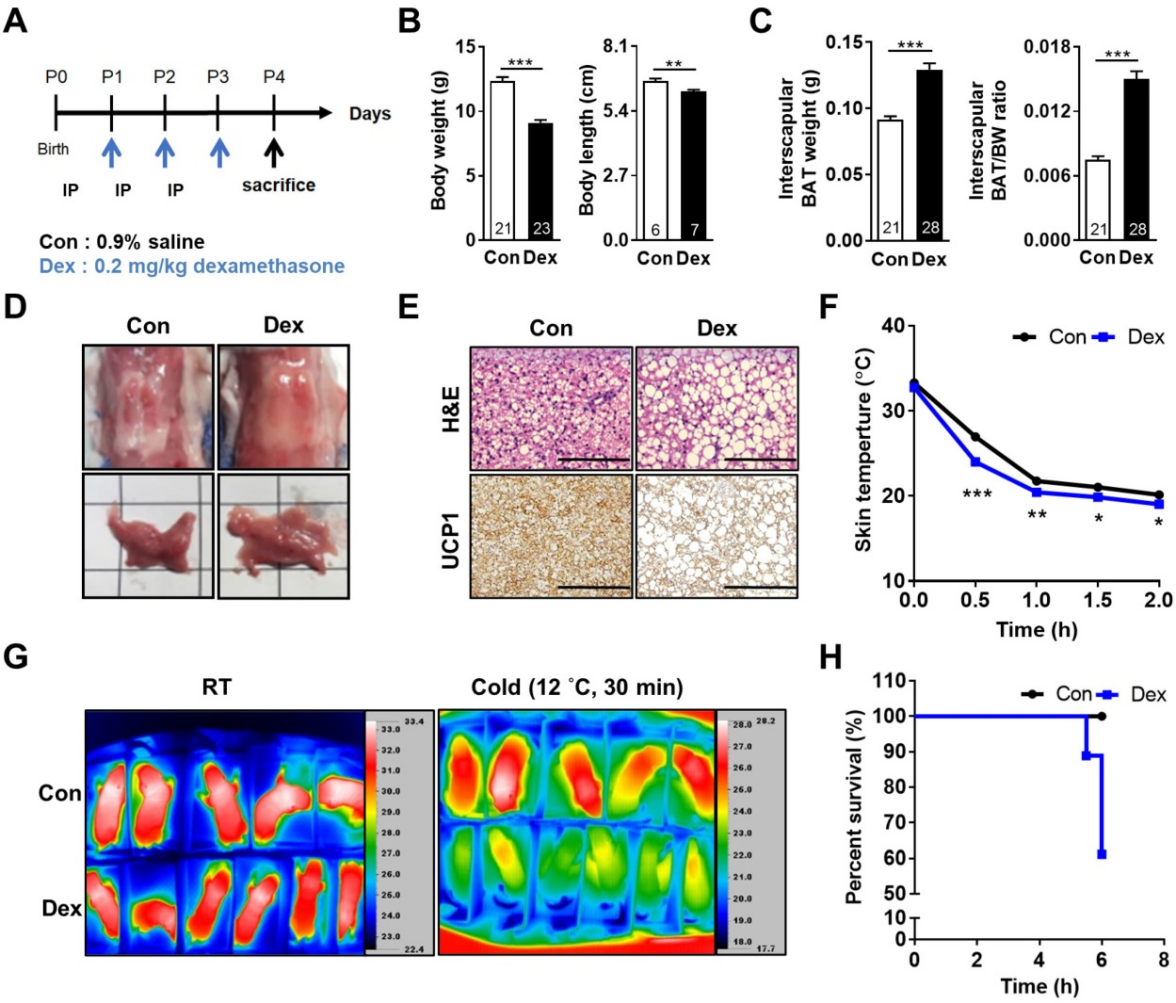
** Effect of Dex on BAT morphology and function of neonatal rats.** (A) Experimental design of Dex treatment in neonatal rats. (B) Body weight and body length (C) Interscapular BAT weight and BAT/body weight ratio. Numbers of rats in each group were indicated within bars. (D) Gross appearance of interscapular BAT. Scale = 1 cm (E) H&E (upper panels) and UCP1 immunohistochemical staining (lower panels) of BAT. Scale bar = 400 μm. (F) Back skin temperature during cold challenge (12 °C). Con, n = 5; Dex, n = 6. (G) Infrared thermo-imaging before the beginning of cold challenge (RT) and 30 min after the beginning of cold challenge (Cold). (H) Survival curves of neonatal rats after cold challenge. Data were expressed as mean ± SEM with statistics analyzed by Student's *t*-test. *p < 0.05, **p < 0.01, and ***p < 0.001.

The pregnant Wistar rats from the BioLASCO Experimental Animal Center (Taipei, Taiwan) were housed in an individual cage with free access to water and food. At postnatal day 0 (P0), neonatal rats were randomly assigned into two groups. They received either saline (Con) or Dex (0.2 mg/kg/day, Sigma-Aldrich, St. Louis, MO, USA) via intraperitoneal injection daily from P1 for 3 consecutive days. On P4, all pups were sacrificed with isoflurane (Attane 074416, Panion & BF Biotech, Taipei, Taiwan) (protocol as shown in Figure 1A), and then BAT was harvested and stored in liquid nitrogen. To examine autophagy flux, chloroquine (CQ; 60 mg/kg) and rapamycin (Rapa; 6 mg/kg) were used as co-treatment 20 min before each Dex or saline injection. AICAR (125 mg/kg; twice daily) was given 1 h before Dex or saline administration. All animal experiments were approved by the Institutional Animal Care and Use Committee of the National Cheng Kung University.

### WT-1 preadipocyte culture

Murine WT-1 preadipocyte line was provided from Dr. Yu-Hua Tseng (Joslin Diabetes Center, Harvard Medical School, Boston, MA). The cells were maintained in 10% FBS of DMEM at 37 °C in 5% CO_2_. To initiate the differentiation process, cells were cultured with induction medium (20 nM insulin, 1 nM triiodothyronine (T3), 1 μM Dex, 1 mM isobutylmethylxanthine (IBMX), 0.125 mM indomethacin in 2% FBS of DMEM) for two days. Then, they were changed into differentiation medium (20 nM insulin, 1 nM triiodothyronine (T3) in 2% FBS of DMEM) every other day and were fully differentiated on day 8.

### Skin temperature and cold tolerance

P4 pups (Con and Dex) were exposed to 12 °C for 6 h to test their cold tolerance 32. Their back skin temperature (focused on the area of interscapular BAT) was monitored and recorded with an infrared ray-based thermal tracer (H2640, Avio, Tokyo, Japan), and analyzed by a software (TAS24, Ching Hsing Computer-Tech Ltd., Taiwan). Digital information was processed by NIH Image 1.55 software.

### RNA isolation and real-time PCR

RNA was extracted with REzol (Protech Technology, London, UK). RNA was dissolved in nuclease-free water and quantified using a Nanodrop ND 2000 (ThermoFisher Scientific, Waltham, MA, USA). Levels of mRNA were analyzed with SYBR green-based real-time quantitative PCR assays (Applied Biosystems, Darmstadt, Germany). Primer sequences are listed in Table S1.

### Western blot

BAT was placed in RIPA buffer containing protease inhibitor cocktail and homogenized. After centrifugation, the resulting supernatant was determined for protein concentration using a protein assay kit (Bio-Rad Laboratories, Hercules, CA, USA). Samples were mixed with SDS loading buffer, boiled, electrophoresed in 10% SDS-PAGE gels, and then transferred to PVDF membranes. Membranes were blocked with blocking buffer for 1 h at room temperature and incubated with primary antibodies (Table S2). After washing, the membranes were incubated with horseradish peroxidase-conjugated secondary antibodies. Immunoreactive protein detection was performed with an enhanced chemiluminescence detection system (PerkinElmer, Waltham, MA, USA).

### Oxygen consumption assay

XF24 extracellular flux (Seahorse Biosciences) was used for the experiment. Mature differentiated WT-1 adipocytes were seeded to XF24 cell culture microplate and incubated for 24 h to ensure attachment. Next day, the cells were treated with different Dex concentrations for 24 h. Before the assay, the cell medium was changed to assay medium (DMEM without sodium bicarbonate) for 15 min at 37 °C without CO_2_. Oxygen consumption rate (OCR) was examined through the sequential injections of oligomycin (4 μM), carbonyl cyanide 4-(trifluoromethoxy) phenylhydrazone (FCCP, 0.2 μM), and rotenone (1 μM).

### Mitochondrial isolation

Tissues in isolation buffer (250 mM sucrose, 0.5 mM EGTA, and 5 mM HEPES, pH 7.4) were homogenized, and centrifuged at 500 ***g*** for 10 min twice. The post-nuclear supernatant as the cytosolic fraction was removed and then centrifuged at 18,000 ***g*** for 25 min. The pellet was resuspended in 20% sucrose, 10 mM Tris, and 0.1 mM EDTA, and centrifuged at 18,000 ***g*** for 30 min. The pellet was then resuspended in 50 μl of isolation buffer as the mitochondrial fraction and determined for protein concentration (Bio-Rad Laboratories, Hercules, CA, USA).

### Electron Microscopy

BAT was immersion-fixed in 0.1 M phosphate buffer (pH 7.3), followed by 2.5% glutaraldehyde and 2% paraformaldehyde in 0.1 M sodium cacodylate buffer. BAT was sliced into 1-mm^3^ cubes and fixed overnight at 4 °C. Sections were examined under transmission electron microscopy (H7600, Hitachi, Tokyo, Japan) at the indicated magnification. Mitochondrial structure, including y/x axis length ratio and length, and cristae numbers were measured in more than 100 mitochondria per rat using ImageJ software. Mitochondrial number and lipid droplets were calculated from 3 fields per rat.

### Immunofluorescent staining and confocal microscopy

BAT was fixed in 4% paraformaldehyde embedded in paraffin and sectioned. After deparaffination, samples were retrieved with citrate buffer (pH 6.0) for 15 min at 80 °C. Then, samples were blocked with 3% BSA blocking buffer for 1 h and incubated with primary antibodies (Table S2) in blocking buffer for overnight at 4 °C, and then incubated with the secondary antibody for 1 h. The specimens were examined with a confocal laser scan fluorescence-inverted microscope (FV1000, Olympus, Tokyo, Japan).

### Immunohistochemistry and H&E stain

BAT was fixed in 4% paraformaldehyde embedded in paraffin and sectioned. After deparaffination, samples were blocked hydroperoxides by 3% H_2_O_2_ solution for 30 min and washed with 1 X PBS three times. Samples were incubated with primary antibodies (Table S2) for overnight at 4 °C and then incubated with the secondary antibody for 1 h. The nucleus was stained by hematoxylin for 2 min. The protein was detected via AEC staining kit (Sigma-Aldrich, St. Louis, MO, USA). For histology, tissue sections were stained by hematoxylin and eosin (H&E). The specimens were examined via a microscope (BX51, Olympus, Tokyo, Japan).

### Patients

We performed a retrospective cohort study. Preterm infants born between January, 2014 to December, 2016, who received systemic corticosteroid (Dex or hydrocortisone) via intravenous route for more than three days were recruited as treatment group; Each patient in the treatment group was matched with two controls born in the same year based on gender and gestational age (± 2 weeks). One of the patients within the treatment group was matched with only one control due to data availability. We compared daily diurnal temperature variation (daily highest minus lowest temperature) between treatment group (during corticosteroid administration) and control group (during the same postnatal age). Mann-Whitney U test was used for data analysis. The institutional review boards at National Cheng Kung University Hospital had approved the study (IRB number B-ER-105-376).

### Statistical analysis

Results were expressed as mean ± SEM. Statistical analyses were conducted by Student's *t* test or one-way ANOVA followed by Fisher's least significant difference comparison test. Differences were considered to be statistically significant at *p* < 0.05.

## Results

### Dex induced growth retardation, BAT whitening and cold intolerance in neonatal rats

At postnatal day 0 (P0), neonatal rats were randomly assigned into two groups. They received either saline (Con) or Dex (0.2 mg/kg/day) via intraperitoneal injection daily from P1 for 3 consecutive days. On P4, all pups were sacrificed (Figure 1A). Dex-treated rat pups showed significantly decreased body weight (BW) and body length but increased interscapular BAT weight and BAT/BW ratio as compared with Con (Figure 1B-C). No gender difference was found (Figure S1A-C). The BAT in Con group presented with its typical reddish-brown color, while the BAT of Dex-treated group appeared hypertrophic and paler in color (Figure 1D). Microscopically, it revealed abundant accumulation of lipid droplets (Figure 1E). To further examine the function of BAT, we exposed these P4 pups (Con and Dex) to 12 °C environment for 6 h. We recorded the change of back skin temperature (the location of interscapular BAT) of rat pups every 30 min with infrared camera. Compared to Con, Dex-treated rats showed steeper drop of body temperature and significantly lower survival rate (60% vs 100%) after cold exposure (Figure 1F-H).

### Dex downregulated UCP1 with compensatory increases in browning and cAMP/PKA pathways

**Figure 2:**
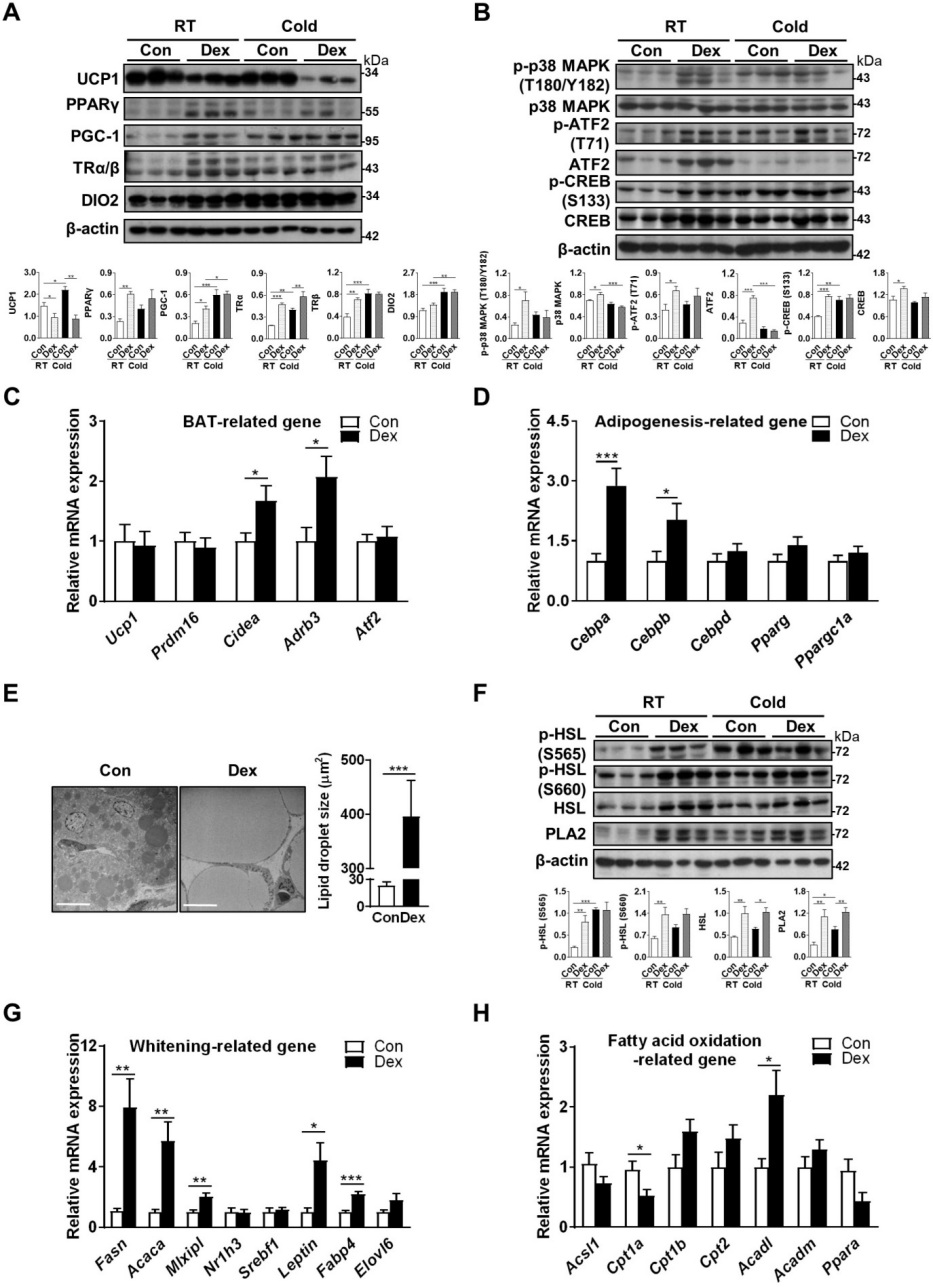
** Effect of Dex on BAT browning, thermogenic pathways and lipid metabolism.** Immunoblot analysis on (A) UCP1 protein, transcriptional factors and cofactors for production of UCP1, and (B) the norepinephrine-stimulated cAMP-PKA signaling pathway under room temperature (RT) or cold challenge (Cold) for 6 h at P4. Each band represents a tissue extract from a single neonatal rat. Expression of (C) UCP1 and transcriptional factors/markers of BAT differentiation (n = 10-13) and (D) adipogenesis-related genes (n = 10-12) in the BAT of Dex-treated and Con neonatal rats. (E) Electron microscopic images and quantification of lipid droplet in the BAT of Dex-treated neonatal rats. The images were obtained at 1200× magnification. Scale bar = 10 μm. (F) Immunoblot analysis on lipolysis-related protein under RT and Cold. Expression of (G) BAT whitening-related genes (n = 10-13) and (H) fatty acid oxidation-related genes (n = 10-13). mRNA levels were expressed relative to average expression in the control rats. Data were expressed as mean ± SEM and statistics were calculated by Student's *t*-test in (C, D, E, G, and H). For quantification of immunoblot analysis, the intensities of bands quantified densitometrically relative to the control at RT were expressed as the bar graph with statistics analyzed by one-way ANOVA in (A, B, and F). *p < 0.05, **p < 0.01, and ***p < 0.001.

Next, we explored the mechanism by which Dex attenuated BAT function. UCP1 protein level was significantly decreased in Dex-treated BAT (lane 4~6 vs lane 1~3, Figure 2A). To reflex the total thermogenic capacity of an intact animal, we calculated total UCP1 protein level, obtained from the data of quantified immunoblots (representing levels of UCP1 protein per mg tissue protein) multiplied by the total protein content in the tissue, which was also significantly decreased after Dex treatment (Figure S2), compatible with phenotypic cold intolerance. Transcriptional factors and cofactors for production of UCP1, including PPAR-γ, PGC-1α, and thyroid receptors TRα/β and DIO2, which activates local T3, were increased after Dex treatment (lane 4~6 vs lane 1~3, Figure 2A). We then examined the norepinephrine-stimulated cAMP-PKA signaling pathway, which is the main signaling pathway responsible for UCP1 production. Phosphorylations of p38 MAPK (T180/Y182), ATF2 (Τ71), and CREB (S133) were up-regulated (lane 4~6 vs lane 1~3, Figure 2B).

To address the contribution of the upstream PKA pathway in cold intolerance after Dex treatment, we performed cold challenge at P4 and examined PKA and thermogenic pathways in BAT by immunoblotting. Our results showed that cold challenge in Con group elicited up-regulation of proteins for thermogenic program (PGC-1 in Figure 2A), thyroid hormone signaling pathway (TRα/β and DIO2 in Figure 2A), and PKA signaling pathway (p-p38, p-ATF2 and p-CREB in Figure 2B). Interestingly however, cold challenge in Dex group failed to induce further increases of these protein levels. Instead, we found that cold challenge decreased UCP1 and ATF2, as well as phosphorylation of p38, in Dex group. These results suggest that upregulation of upstream PKA pathway and thermogenic program in Dex group under room temperature might be compensatory mechanism in response to the defective thermogenesis.

However, gene transcripts of *Ucp1* and other markers of BAT differentiation, including *Prdm16* and *Atf2*, were not significantly different between Dex and Con groups. Gene transcripts of *Cidea* and *Adrb3* (β3-adrenoceptor) were increased in Dex group (Figure 2C). For adipogenesis-related genes, Dex treatment significantly increased the transcription of *Cebpa* and *Cebpb*, despite no significant elevation on the expression of *Cebpd, Pparg* and *Ppargc1a* (Figure 2D). Overall, these findings suggested that BAT dysfunction after Dex treatment was accompanied by compensatory increases in BAT differentiation and cAMP-PKA thermogenic pathways.

### Dex induced neonatal BAT whitening by enhancing lipogenesis

We found that BAT of Dex-treated rat pups showed morphological whitening with abundant lipid droplet accumulation (Figure 1D-E). Under electron microscope, we observed many large, unilocular lipid droplets in the BAT of Dex-treated rat pups (Figure 2E). These morphological changes may result from either increased lipogenesis, decreased lipolysis or decreased β-oxidation. Total and phosphorylated HSL (S565 and S660), the key enzymes involved in lipolysis, were elevated in Dex group. In addition, the level of PLA2 (phospholipase A2), which generates long-chain fatty acids (LCFA) in the inner membrane of mitochondria required for proton transport through UCP1 (UCP1 is a H^+^/LCFA^-^ symporter), was also higher in Dex group, suggesting a robust lipid mobilization for thermogenesis. Cold challenge up-regulated phosphorylated HSL (S565) and PLA2 in Con group; however, in Dex group, cold challenge failed to induce further increases of these proteins (Figure 2F).

For whitening-related genes, transcription of key enzymes involved in lipogenesis, FAS (fatty acid synthase, *Fasn*) and ACC (acetyl-CoA carboxylase, *Acaca*), were substantially increased in Dex group (Figure 2G). Among transcriptional factors mediating lipogenesis, carbohydrate-responsive element-binding protein (ChREBP, *Mlxipl*) was elevated in Dex group; whereas liver x receptor (LXR, *Nr1h3*) and sterol regulatory element-binding protein 1 (SREBP1, *Srebf1*) were not different between the two groups. White adipocyte marker leptin (*Leptin*) was also substantially increased. In addition, Dex treatment significantly increased the expression of *Fabp4* (fatty acid-binding protein 4) and tended to increase *Elovl6* (elongation of very long-chain fatty acids protein 6), which are enzymes participating in fatty acid binding and *de novo* lipogenesis, respectively, and are essential for BAT whitening (Figure 2G). For the enzymes involved in fatty acid oxidation, Dex treatment decreased mRNA level of *Cpt1a*, one of the iso-form enzymes of carnitine palmitoyl transferase 1 (CPT1) responsible for the first rate-limiting step of fatty acid oxidation, and *Ppara* (peroxisome-proliferator activated receptor alpha, PPAR-α) (Figure 2H). The expression of *Acadl*, long-chain acyl-CoA dehydrogenase (LCAD) for breaking down of LCFA, was significantly increased. The mRNA levels of *Cpt1b*. *Cpt2* and *Acadm* (medium-chain acyl-CoA dehydrogenase, MCAD) showed increasing trends. Altogether, these results suggested that Dex induced BAT whitening by enhancing lipogenesis and dysregulating fatty acid oxidation.

### Dex skewed mitochondrial fission-fusion balance in neonatal BAT

**Figure 3:**
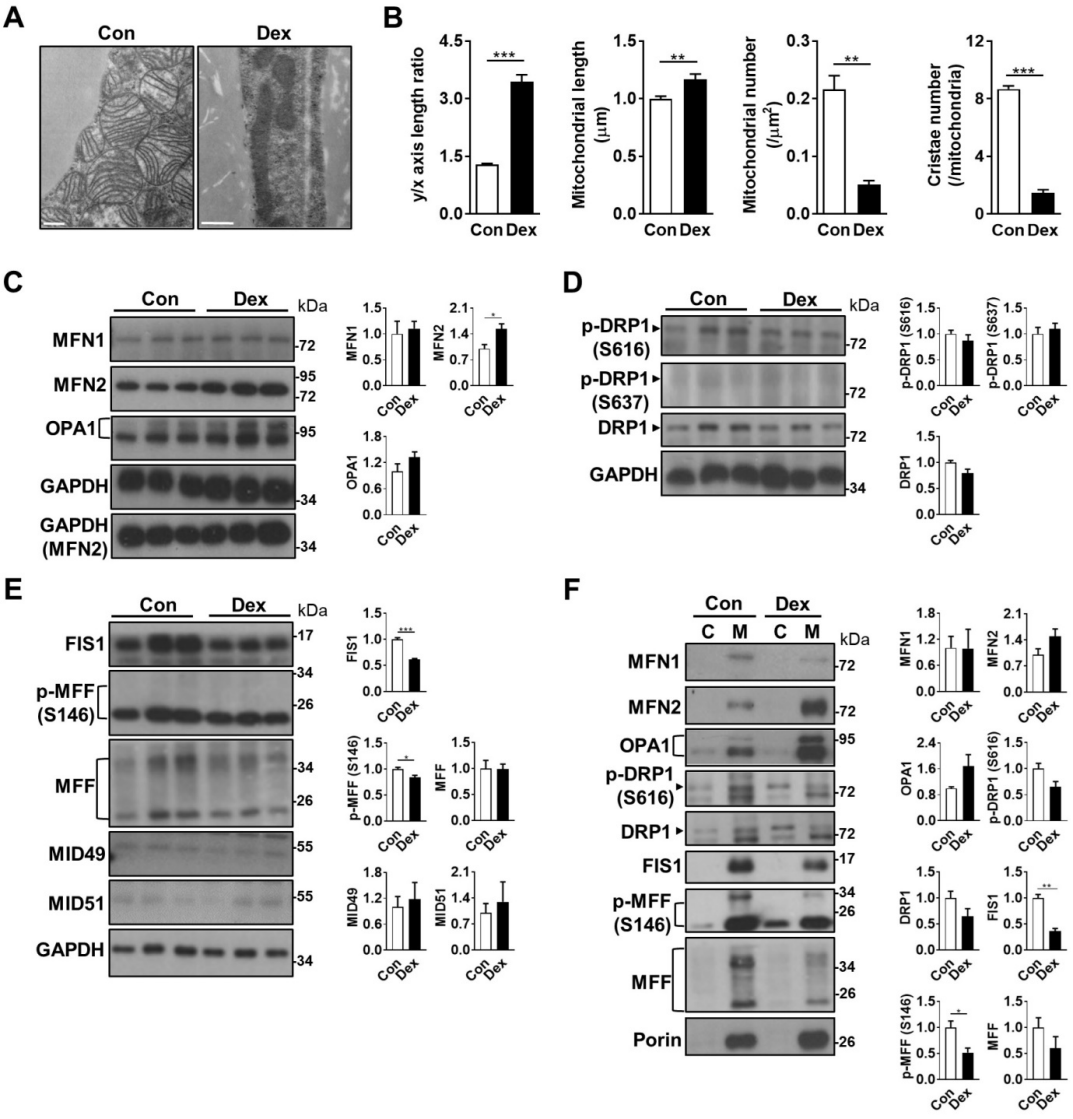
** Effect of Dex on BAT mitochondrial fusion/fission machinery.** (A) Electron microscopic images and (B) quantitative analysis of mitochondrial geometry (n = 63) and cristae number (n = 17). Scale bar = 500 nm. Immunoblot analysis on (C) mitochondrial fusion and (D & E) fission-related proteins in the total cell lysate, as well as (F) from the cytosolic **(C)** and mitochondrial **(M)** fractions, of BAT. Each band in Fig. 3F represented a tissue extract from the cytosolic **(C)** and mitochondrial **(M)** fractions, of BAT of a single neonatal rat. Representative bands are selected from three independent experiments with quantitation results. Data were expressed as mean ± SEM and statistics were calculated by Student's *t*-test. For quantification of immunoblot analysis, the intensities of bands quantified densitometrically relative to the control were expressed as the bar graph with statistics analyzed by Student's *t*-test in (C, D, E, and F). *p < 0.05, **p < 0.01, and ***p < 0.001.

Because morphological whitening and cold intolerance were both suggestive of impaired BAT function, we hypothesized that mitochondria, the key machinery in BAT for UCP1 function, might be damaged after Dex treatment. Under electron microscope, we found that the mitochondria in BAT after Dex treatment were elongated (reflected by y/x axis length ratio and mitochondrial length), fewer in number, more electron-dense and lacked normal alignment and number of cristae (Figure 3A-B). The expression of genes related to mitochondrial biogenesis, including PCC-1α *(Ppargc1a)*, mitochondrial transcription factor A (*Tfam*), and estrogen-related receptor alpha (*Esrra*), were not significantly different between Dex and Con groups (Figure 2D and S3A). For molecules involved in mtDNA replication and repair, the expression of single-stranded DNA binding protein 1 (*Ssbp1*), but not polymerase gamma 2 (*Polg2*), was significantly increased after Dex treatment. Transcripts of mitochondrial electron transport chain (ETC) subunits, including complex I (NADH dehydrogenase subunits 1 (*Nd1*) and *Nd3*), complex III (cytochrome b, *Cytb*), complex IV (cytochrome oxidase subunit 3, *Cox3*), and complex V (*Atp6*), were significantly increased after Dex treatment (Figure S3B). Altogether, these results suggested that Dex did not attenuate mitochondrial biogenesis or transcription of ETC subunits.

Mitochondrial fission and fusion play critical roles in maintaining functional mitochondria. Because the mitochondria in Dex group were elongated in shape, we examined the balance of mitochondrial fusion and fission machinery. First, we examined mitochondrial fusion proteins, and found that after Dex treatment, the level of mitofusin 2 (MFN2) was significantly increased in the tissue lysate; optic atrophy 1 (OPA1) protein also showed a trend of increase; while the level of MFN1 protein was similar (Figure 3C). For mitochondrial fission proteins, the level of dynamin-related protein 1 (DRP1) showed a decreasing trend in Dex group but did not reach statistical significance (Figure 3D). The level of p-DRP1 (S616), which promotes fission, was similar between the two groups; the level of p-DRP1 (S637) protein, which induces detachment of DRP1 from mitochondria and inhibits fission, was barely detectable in both groups. The levels of two DRP1 receptor proteins, fission related protein-1 (FIS1), and phosphorylation of mitochondrial fission factor (MFF) at S146, were both significantly decreased; No significant difference was found in the level of total MFF (Figure 3E). The levels of MID49 and MID51, another two DRP1 receptors, were barely detectable (Figure 3E). Immunoblotting of subsequent mitochondrial fraction showed increased translocation of fusion proteins (MFN2 and OPA1) and decreased translocation of fission proteins (p-DRP1 S616, DRP1, FIS1, p-MFF S146 and MFF), to the mitochondria (Figure 3F). In conclusion, these results suggested that the abundance of elongated mitochondria in Dex-treated BAT may result from the skewed fusion-fission balance toward increased fusion.

### Dex impaired autophagosome degradation in neonatal BAT

**Figure 4:**
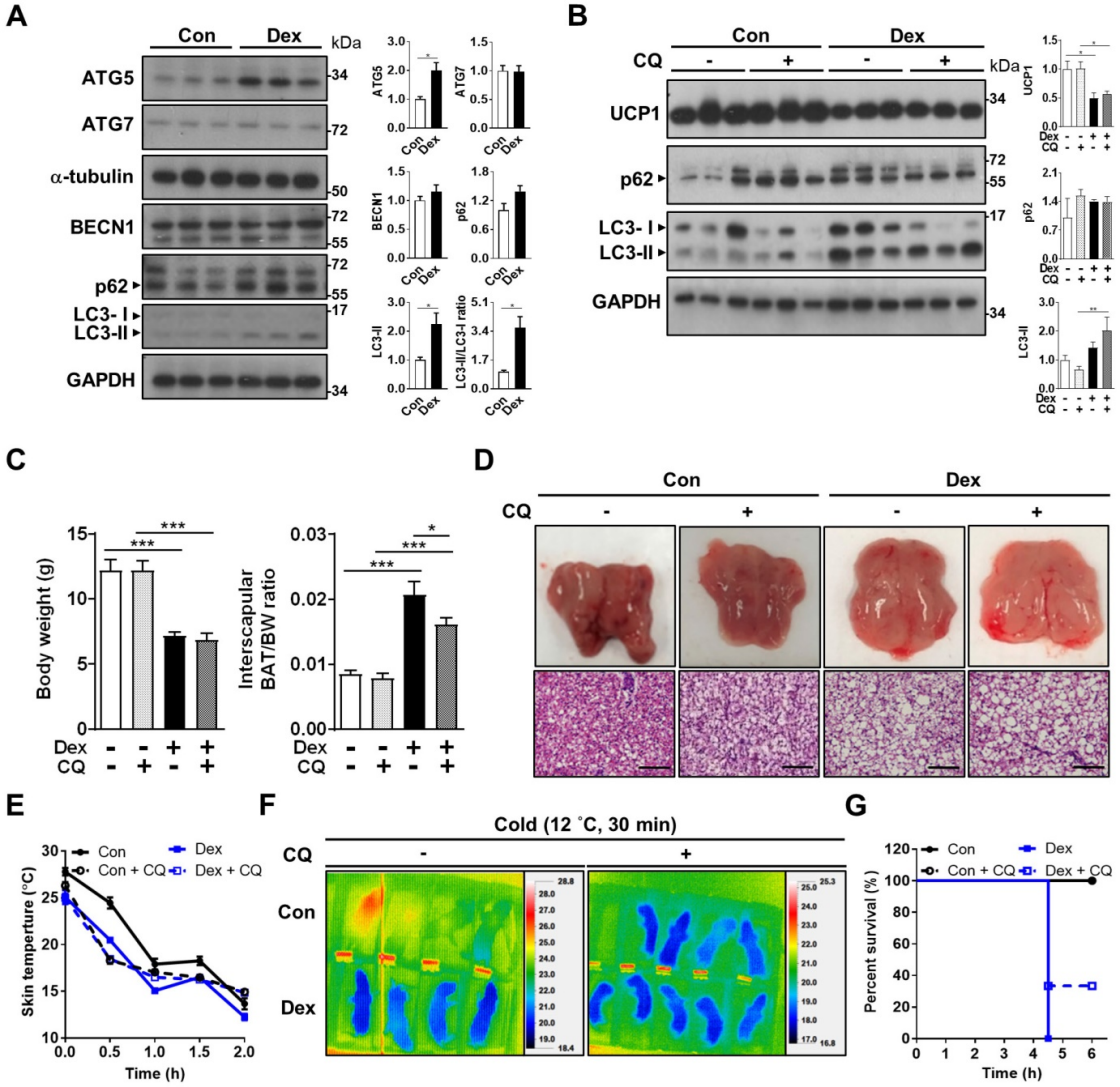
** Effect of Dex on BAT autophagy and co-treatment with CQ.** (A) Immunoblot analysis on autophagy-related proteins. (B) Evaluation of autophagy flux with CQ by immunoblot analysis on UCP1 and autophagy-related proteins. (C) Body weight, BAT/body weight ratio. (D) Gross appearance and H&E stain of interscapular BAT. Con, n = 5; Con+CQ, n = 6; Dex, n = 6; Dex+CQ, n = 7. Scale bar = 200 μm. (E) Infrared thermo-imaging at 30 min after the beginning of cold challenge (Cold). (F) Back skin temperature during cold challenge (12 °C). (G) Survival curves of neonatal rats after cold challenge. Con, n = 4; Con+CQ, n = 4; Dex, n = 4; Dex+CQ, n = 5. Data were expressed as mean ± SEM and statistics were calculated by one-way ANOVA. For quantification of immunoblot analysis, the intensities of bands quantified densitometrically relative to the control were expressed as the bar graph with statistics analyzed by Student's *t*-test in (A) and by one-way ANOVA in (B). *p < 0.05, **p < 0.01, and ***p < 0.001.

Since mitochondrial fission is prerequisite for autophagy, we speculated that the disrupted fission-fusion balance after Dex treatment might also influence autophagy flux. Autophagy is critical for maintaining proper cellular and mitochondrial functions, and is induced and required for optimal BAT non-shivering thermogenesis upon cold challenge 33, 34. Therefore, we examined whether Dex treatment affected autophagy flux in BAT. First, we investigated the well-established pathway of ATG (autophagy related-protein)-5 dependent autophagy (conventional autophagy). Protein level of ATG5 was significantly increased after Dex treatment; but ATG7 and Beclin-1 levels were not (Figure 4A). We noted that LC3-II level and LC3-II/I ratios, as well as SQSTM1/p62 level, were increased after Dex treatment. These results suggested that Dex treatment caused either increased autophagosome synthesis or impaired autophagosome degradation. To discriminate between these two possibilities, we then applied chloroquine (CQ), which blocks autophagosome degradation and “clamped” LC3-II degradation, for 3 consecutive days. We found that CQ treatment resulted in higher p62 level in Con group (lane 4~6 vs lane 1~3, Figure 4B), showing the effect of CQ. However, the addition of CQ resulted in limited increased levels of LC3-II and p62 in Dex group (lane 10~12 vs lane 7~9, Figure 4B). These results implicated a likely block in autophagosome degradation by Dex, because CQ cannot have further effect on Dex group 35. Consistently, colocalization of mitochondria and LC3-II increased after Dex treatment but did not further increase after CQ co-treatment, also suggesting impairment in autophagosome degradation (Figure S4A-B). We found that CQ co-treatment failed to reverse Dex-induced stunted growth, BAT hypertrophy (Figure 4C) and whitening (Figure 4D and Figure S5A) and UCP1 level (Figure 4B). Functionally, CQ treatment in Con neonatal rat pups induced an even steeper drop in body temperature than Con rats (Figure 4E-F and Figure S5B). CQ co-treatment also failed to reverse the Dex-induced body temperature drop and lower survival rate after cold exposure (Figure 4E-G). Therefore, we concluded that Dex treatment, though increased autophagosome synthesis, impaired autophagosome degradation. CQ co-treatment failed to rescue Dex-mediated BAT dysfunction in neonatal rats.

### Activation of autophagy by rapamycin failed to reverse the Dex-induced BAT whitening in neonatal rats

**Figure 5:**
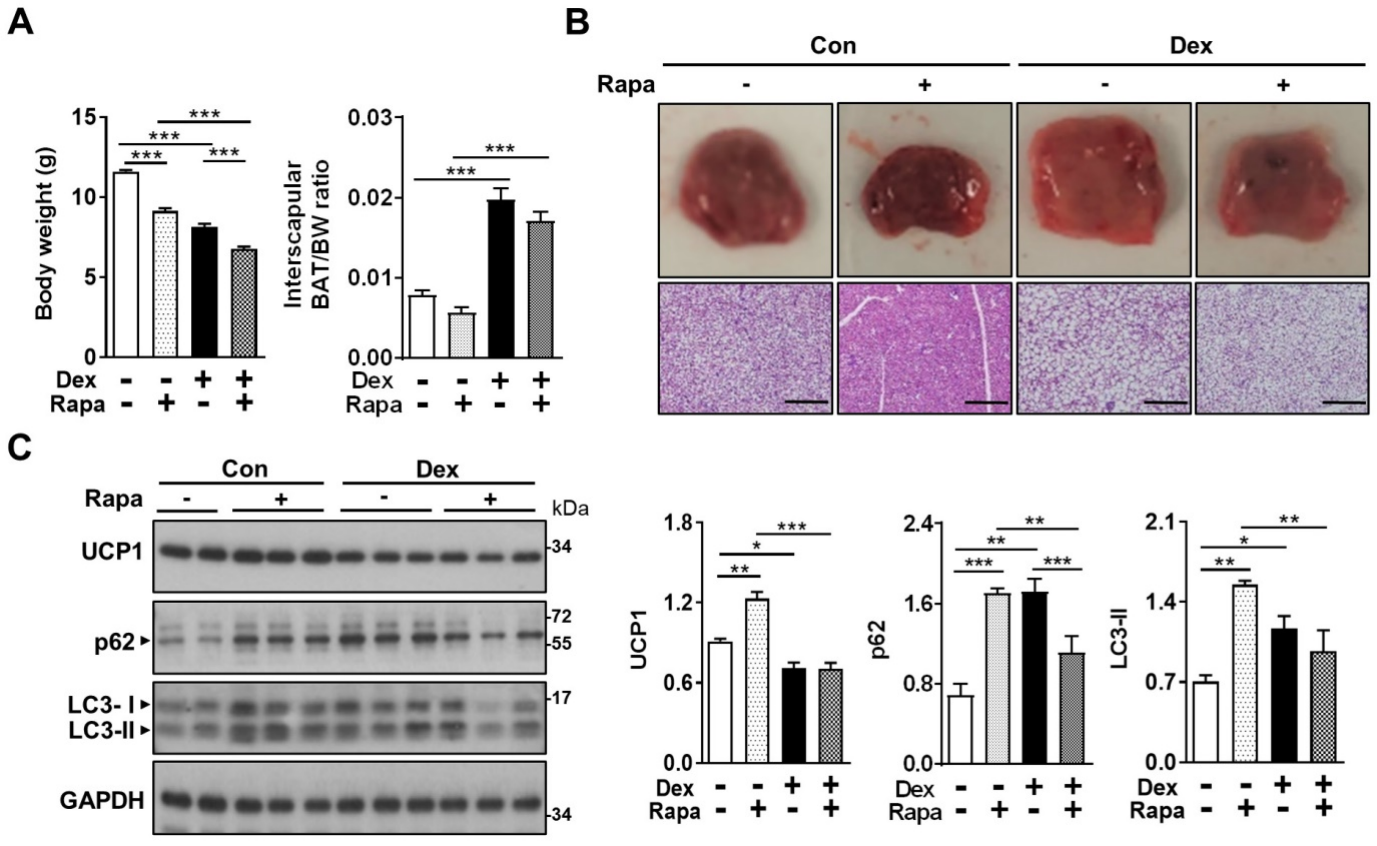
** Effect of Dex on BAT autophagy and co-treatment with Rapa.** Effect of co-treatment with Rapa: (A) Body weight and BAT/body weight ratio. Con, n = 3; Con+Rapa, n = 3; Dex, n = 3; Dex+Rapa, n = 3. (B) Gross appearance and H&E stain of interscapular BAT. (C) Immunoblot analysis of UCP1 and autophagy-related proteins after co-treatment with Rapa. Data were expressed as mean ± SEM and statistics were calculated by one-way ANOVA. For quantification of immunoblot analysis, the intensities of bands quantified densitometrically relative to the control were expressed as the bar graph with statistics analyzed by one-way ANOVA in (C). *p < 0.05, **p < 0.01, and ***p < 0.001.

In the attempt to reverse the impaired autophagy flux induced by Dex, we next tried co-treatment with rapamycin (Rapa), for 3 consecutive days 36. We speculated that Rapa may be able to reverse the effects of Dex by “dredging” the stunted autophagy flux. We found that treatment with Rapa resulted in significant decreases in body weight gain in both Con and Dex groups, reflecting its systemic influence (Figure 5A). BAT/BW ratio was decreased after co-treatment with Rapa in Dex group, indicating that Rapa may partially reverse the Dex-induced BAT hypertrophy (Figure 5A). Grossly, the color of interscapular BAT in both Con and Dex groups were darker after Rapa treatment. Histologically, however, Rapa significantly decreased lipid droplet accumulation significantly only in Con group (Figure 5B). We did observe some microscopic fields with decreased size of lipid droplets in Dex+Rapa group; however, overall whitening of BAT was not reversed by Rapa. Immunoblotting showed Rapa treatment increased LC3-II and p62 levels in Con group, suggesting Rapa increased synthesis of autophagosome (lane 3~5 vs lane 1~2, Figure 5C). Dex treatment alone showed comparable accumulation of LC3-II and p62 with Con+Rapa (lane 6~8 vs lane 3~5, Figure 5C), confirming that Dex increased autophagosome synthesis. Rapa co-treatment attenuated the increased LC3-II and p62 levels, but did not recover the decreased UCP1 level (lane 9~11 vs lane 6~8, Figure 5C). These results suggest that activation of autophagy alone is not sufficient to rescue Dex-mediated BAT dysfunction in neonatal rats.

### AMPK activator attenuated UCP1 decrease *in vitro*

**Figure 6:**
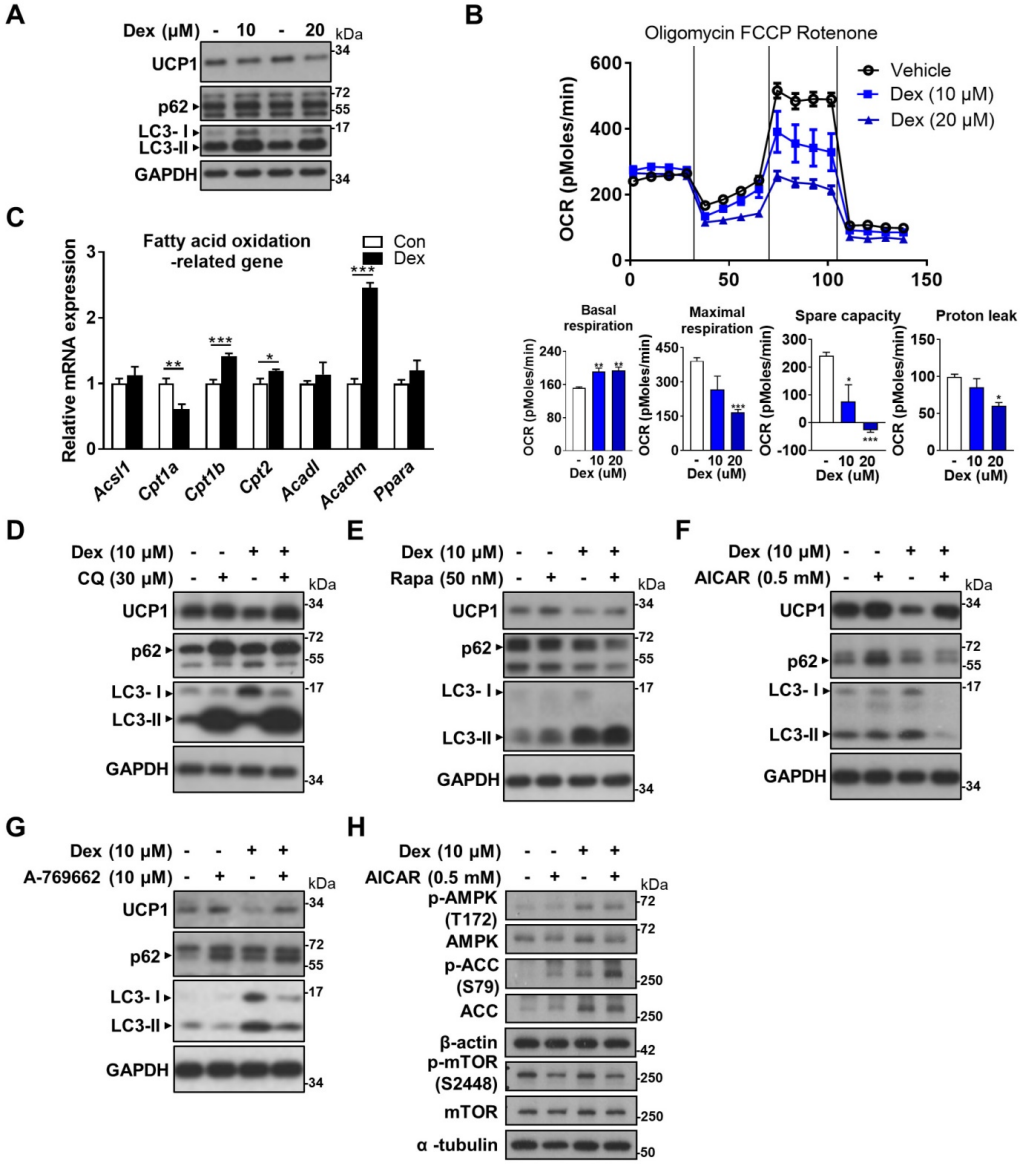
** Effect of Dex on WT-1 brown adipocytes.** (A) Immunoblot analysis on UCP1 and autophagy-related proteins. (B) Analysis of oxygen consumption rate (OCR) with Seahorse analyzer. (C) Expression of fatty acid oxidation-related genes (n = 6). mRNA levels were expressed relative to average expression in the control group. Investigation of the effect of Dex on autophagy flux with (D) CQ and (E) Rapa. Immunoblots of UCP1 and autophagy-related proteins after co-treatment with (F) AICAR and (G) A-769662. (H) Immunoblots of AMPK pathway after co-treatment with AICAR. Data were expressed as mean ± SEM and statistics were calculated by one-way ANOVA in (B) and Student's *t*-test in (C). *p < 0.05, **p < 0.01, and ***p < 0.001.

In search of a potential rescue, we looked to our *in vitro* model for answer by using differentiated WT-1 brown adipocytes. Dex treatment resulted in decreased UCP1 and increased LC3-II proteins, compatible with our findings *in vivo* (Figure 6A). Seahorse analysis showed a dose-dependent decline in oxygen consumption rate (OCR) in maximal respiration, spare capacity and proton leak as the dose of Dex increased (Figure 6B). Since proton leak is largely contributed by UCP1 in brown adipocytes, the decrease in proton leak is compatible with the finding of decreased UCP1 protein level after Dex treatment in western blot. We next examined how Dex affects fatty acid oxidation. Dex treatment significantly decreased mRNA level of *Cpt1a*; while it increased mRNA levels of *Cpt1b* and *Cpt2* as well as *Acadm (*medium-chain acyl-CoA dehydrogenase, MCAD) that is important for breaking down of medium-chain fatty acids (Figure 6C).

CQ and Rapa co-treatments both further increased LC3-II levels compared with Dex alone (Figure 6D-E). UCP1 protein level was slightly increased after CQ treatment, but was not recovered after Rapa treatment. Interestingly, co-treatment of Dex with two direct AMP-activated protein kinase (AMPK) activators, AICAR and A-769662, on the contrary, both significantly decreased LC3-II levels (Figure 6F-G). In addition, UCP1 protein levels were significantly recovered in Dex+AICAR and Dex+A-769662 groups compared with the respective Dex alone group. To clarify whether Dex selectively affects AMPK and mTOR pathways to target autophagy, we examined these pathways *in vitro*. We found that Dex treatment increased phosphorylation of AMPK and ACC (Figure 6H, lane 3 vs lane 1). In contrast, Dex treatment did not alter phosphorylation of mTOR S2448. These results suggest that Dex selectively affects AMPK pathway. AICAR co-treatment increased phosphorylation of AMPK and ACC in both Con+AICAR (lane 2) and Dex+AICAR (lane 4) groups compared with Con (lane 1) and Dex (lane 3) groups, respectively, which can serve as the control for AMPK activation. AICAR co-treatment also attenuated mTOR activity as expected, reflected by decreased phosphorylation of mTOR S2448 (lane 2 vs lane 1; lane 4 vs lane 3).

### AMPK activator partially rescued the Dex-induced BAT whitening *in vivo*

**Figure 7:**
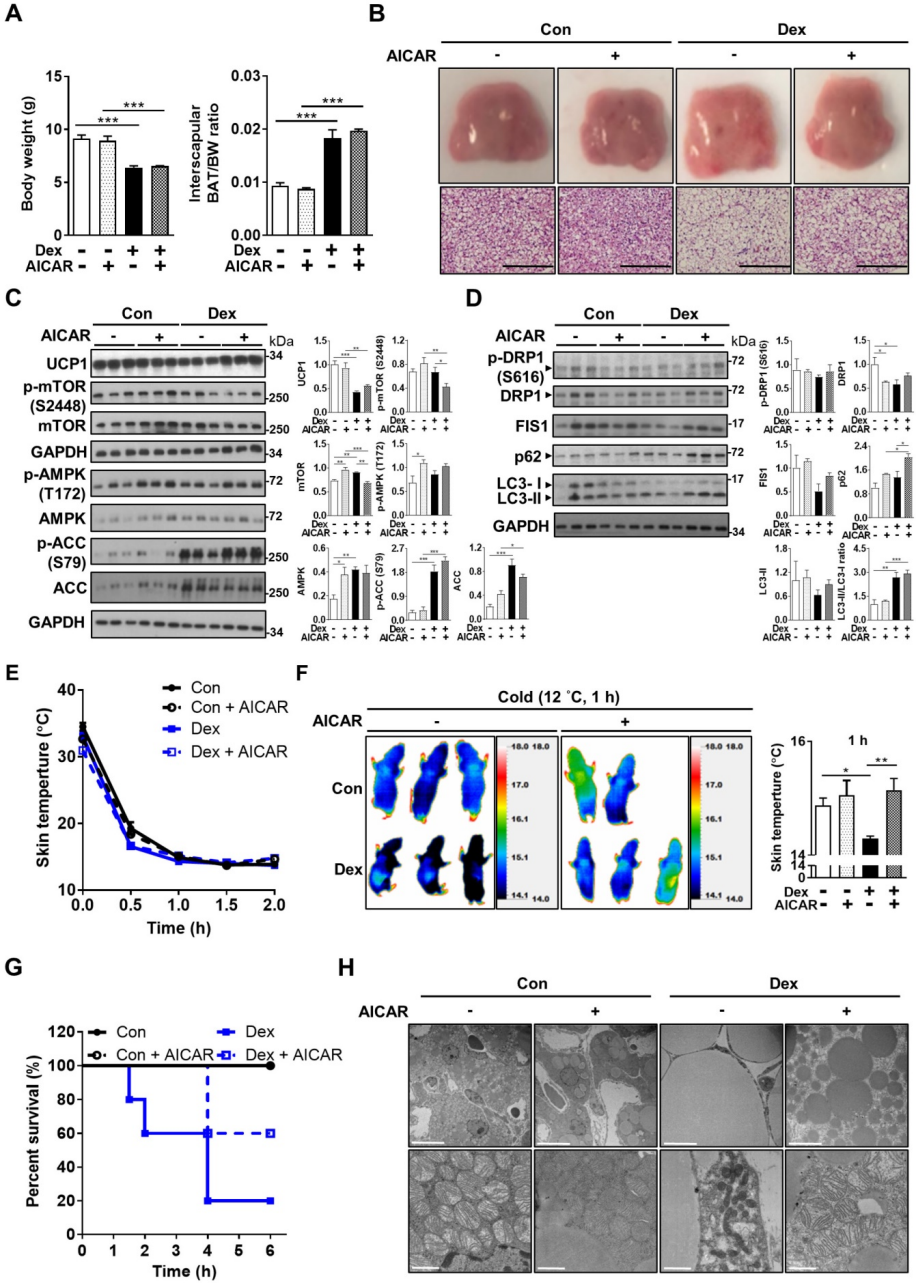
** Effect of AMPK activator co-treatment in neonatal rats.** (A) Body weight and BAT/body weight ratio, Con, n = 3; Con+AICAR, n = 3; Dex, n = 4; Dex+AICAR, n = 4. (B) Gross appearance and H&E stain of interscapular BAT, Con, n = 5; Con+AICAR, n = 6; Dex, n = 6; Dex+AICAR, n = 7. Scale bar = 200 μm. Immunoblot analysis on (C) UCP1 protein and AMPK signaling pathway and (D) fission proteins and autophagy-related proteins after co-treatment with AICAR. (E) Infrared thermo-imaging at 1 h after the beginning of cold challenge and (F) back skin temperature during cold challenge (12 °C). (G) Survival curves of neonatal rats after cold challenge. Con, n = 6; Con+AICAR, n = 5; Dex, n = 5; Dex+AICAR, n = 5. (H) Electron microscope images. Scale bar = 10 μm in upper panels and 1 μm in lower panels. Data were expressed as mean ± SEM and statistics were calculated by one-way ANOVA. For quantification of immunoblot analysis, the intensities of bands quantified densitometrically relative to the control were expressed as the bar graph with statistics analyzed by one-way ANOVA in (C and D). *p < 0.05, **p < 0.01, and ***p < 0.001.

Based on these *in vitro* findings, we next tried co-treatment with AICAR in neonatal rats. Interestingly, we found that although Dex-induced BAT hypertrophy remained (Figure 7A), AICAR treatment partially reversed the Dex-induced BAT whitening (Figure 7B). Furthermore, AICAR co-treatment modestly attenuated Dex-induced UCP1 down-regulation (Figure 7C, lane 10~12 vs lane 7~9). Dex treatment, compared with Con, increased phosphorylation of AMPK and ACC, but did not alter phosphorylation of mTOR S2448 (Figure 7C, lane 7~9 vs lane 1~3), suggesting that Dex selectively affects AMPK pathway. AICAR co-treatment further increased Dex-induced phosphorylation of AMPK and ACC, serving as the control for AMPK activation (Figure 7C, lane 10~12 vs lane 7~9). DRP1 and FIS1 protein levels were also increased (Figure 7C, lane 10~12 vs lane 7~9), supporting that AICAR increased mitochondrial fission. In contrast to the finding of reduced LC3-II and p62 protein levels *in vitro*, AICAR co-treatment in Dex group increased LC3-II and p62 protein levels *in vivo* (Figure 7C, lane 10~12 vs lane 7~9).

Consistently, rat pups in the Dex+AICAR group had significantly higher skin temperature under cold challenge compared with those in the Dex group (Figure 7E-F). Overall survival was also improved in Dex+AICAR group compared with Dex group (60% vs 20%) (Figure 7G). Under electron microscope, AICAR treatment reversed the size of lipid droplets in the BAT (Figure 7H). Furthermore, the mitochondria within the BAT of Dex+AICAR group exhibited round shapes with abundant, dense and aligned cristae, which are in stark contrast to the mitochondria of Dex group. These results suggested that AMPK activators not only activated autophagy but also regulated mitochondrial fission, therefore capable of alleviating Dex-mediated BAT dysfunction in neonatal rats.

### Postnatal systemic corticosteroid administration was associated with greater temperature fluctuation in preterm infants

We investigated whether postnatal exposure to systemic corticosteroid (intravenous Dex and hydrocortisone) in preterm infants disrupted BAT function, leading to temperature instability. Because preterm infants are nursed in incubators equipped with servo-controlled air temperature, based on the difference between the baby's skin temperature and the preset target temperature, it is unlikely that hypothermia would occur. We speculate that BAT dysfunction may be reflected by greater fluctuation of the baby's body temperature within the normal range.

Premature infants who received systemic corticosteroid for lung inflammation were included. Those with possible infection (including statements in medical records, fever ≥ 38 °C, CRP ≥ 10 mg/L or positive blood culture) during treatment or with hypo-/hyperthyroidism were excluded. Finally, eleven infants who received corticosteroid treatment and 21 gestational age (GA) (± 2 weeks)- and sex-matched controls were eligible for final data analysis. Eight cases received intravenous hydrocortisone and three cases received intravenous Dex treatment. We retrieved 993 and 1775 body temperature records from medical records in the treatment and control groups respectively. The median duration of treatment was 13 days (range: 11-16). The median GA of cases was 25 weeks' gestation (range: 22-28 weeks). We observed a statistically higher diurnal temperature variation in the treatment group compared with control (median 0.60 vs. 0.50 °C, *p* = 0.015). (Figure S6A-B). These results indicated that postnatal corticosteroid treatment was associated with greater body temperature fluctuation in preterm infants.

## Discussion

Our study showed that postnatal low-dose Dex exposure caused stunted growth, BAT whitening, and impairment of thermogenesis, leading to cold intolerance in neonatal rat pups. The mitochondria in the Dex-treated BAT were damaged, presenting with electron dense characteristics, elongation in shape and loss of normal cristae pattern. While Dex increased autophagosome synthesis, it impaired autophagosome degradation, leading to the accumulation of damaged mitochondria. Clamping the autophagosome degradation by CQ did not rescue the damage but further worsened the situation by impairing clearance of autophagosome. Autophagy inducer Rapa also failed to rescue the Dex-induced BAT whitening. Nevertheless, we found that AMPK activator, through augmentation of autophagy flux and regulation of mitochondrial dynamics, partially rescued the Dex-induced BAT whitening and decreased UCP1 protein level.

GCs have been shown to inhibit UCP1 transcription and translation 21, 37. However, all of these studies were performed in adult mice or rats. Therefore, the results of these studies cannot be extrapolated to neonates, especially premature infants. As rat and human develop over different embryonic time scales, rat pups on P1 correspond to the human fetus at about week 22 to 24 of gestation 38-41. Therefore, rat pups, which were used in our study, served as an ideal animal model to study human preterm infants.

The effect of corticosteroid on BAT has been dissected in animals with various dosages of regimen, ranging from 0.1-0.5 mg per day lasting 1-6 days 20, 22, 42, 43. Our previous study showed that early administration of Dex (on P1) with Dex as low as 0.2 mg/kg to rat pups resulted in apoptosis of neural progenitor cells in the hippocampus 44. One of the regimens for treatment of BPD with the lowest dose of Dex reported was the “Minidex therapy” (Dex 0.05 mg/kg per day), which was proved to facilitate extubation of mechanically-ventilated infants 45. The human-equivalent dose of 0.2 mg/kg in our animal study is approximately 0.03 mg/kg using allometric scaling 46, which is even lower than Minidex therapy. Thus, our experimental regimen is a reasonable approximate of clinical scenario.

GCs play an important role in the differentiation of brown adipocyte; the basic differentiation cocktail of brown adipocyte consists of Dex 47, 48. However, there is conflicting information on the influences of exogenous GCs on BAT; GCs have been proved to inhibit expression of UCP1 in rodents 20, 21, 49. However, the effect on human BAT remains controversial. For example, acute Dex (1 μM, 5 h) suppressed isoprenaline-stimulated UCP1 mRNA expression* in vitro*, but Dex alone did not affect UCP1 expression 49. In contrast, GC in a high physiological dose and acute exposure (100 nM cortisol *in vitro* and 20 mg prednisolone *in vivo* for 24 h) induced human BAT activation 49. Therefore, marked species-specific differences exist in the effect of GCs on BAT.

Mitochondria play a key role in energy metabolism in brown adipose tissue. Our results showed that after Dex treatment, mitochondria in BAT changed in shape and lost its normal alignment of cristae. Mitochondrial fusion-fission balance was skewed toward increased fusion, characterized by increased OPA1 and MFN2 protein levels and decreased DRP1, FIS1, p-MFF protein levels, after Dex treatment, and these were compatible with the accumulation of elongated mitochondria under electron microscope. This phenomenon can be related to stress (Dex)-induced mitochondrial hyperfusion. Thus, under modest stress (well below those needed to induce apoptosis), partially-damaged mitochondria fuse together to mitigate the cellular burden by optimizing mitochondrial ATP production and to escape from autophagy 50, 51. Another key finding of our study was significant BAT enlargement and whitening after Dex treatment, characterized by accumulation of enlarged lipid droplets. BAT consumes fat to generate heat under cold environment and mitochondrial fatty acid oxidation is indispensable for this process. Although there was discrepancy between *in vivo* and *in vitro* results of the expression profile of fatty acid oxidation-related enzymes*,* we found the expression of *Cpt1a,* the first rate-limiting enzyme of mitochondrial fatty acid oxidation, to be consistently decreased after Dex treatment. Our result is consistent with previous studies, showing that Dex treatment significantly down-regulated the expression of CPT1 in skeletal muscles and cardiomyocytes 52-54. It is reasonable to speculate that the up-regulation of fatty acid oxidation downstream genes (*Acadl* and *Acadm*) may be related to a compensatory effect. Thus, Dex induced BAT whitening partly through a dysregulation in fatty acid oxidation.

Autophagy has been shown to be involved in brown adipocyte differentiation and function 29, 55-57. Dex was reported to induce autophagy in a variety of cells 58-60, yet its effect on autophagy in BAT is still unclear. One recent study by Deng *et al.* showed that Dex induced whitening of BAT via BTG1- and ATG7-dependent autophagy 28. In their study, although inhibition of autophagy by CQ reversed morphological whitening and UCP1 mRNA level, whether it also recovered UCP1 protein or thermogenesis was not demonstrated. In our *in vitro* experiment, CQ co-treatment also slightly recovered Dex-induced decrease of UCP1 protein, somewhat in accordance with their results. However, in our animal model, we showed that although Dex increased autophagosome synthesis, it impaired autophagosome degradation. Therefore, clamping the autophagosome degradation by CQ did not reverse BAT whitening, cold intolerance, and UCP1 protein level. In addition, Deng *et al.* used 8-10 weeks adult mice with 50-fold higher dose of Dex (5 mg/kg), in contrast to using neonatal rat pups with substantially lower dosage (0.2 mg/kg) in the current study. Therefore, our study better corresponded to the clinical scenario of preterm infants receiving low-dose Dex soon after birth.

We further tested whether “dredging” the clogged autophagy flux by Rapa could rescue the adverse effects of Dex on BAT. To our disappointment, Rapa only resulted in minimal improvement of morphological whitening. We speculated that its positive effects on autophagy flux were offset by its disturbance of multiple pathways, including protein synthesis and mitochondrial biogenesis 36, 61. Previous studies showed that mTORC1 is essential for BAT adaptation to cold and β-adrenergic stimulation through promoting mitochondrial biogenesis 62, 63. Rapa was also shown to inhibit UCP1 protein translation 64. Thus, inhibition of mTORC1 resulted in a complicated outcome for thermogenesis and BAT function. Therefore, we did not perform cold challenge test on the Rapa group because we are questionable about its potential in rescuing the Dex-induced BAT damage, particularly accompanied by several severe side effects. We then demonstrated that AMPK activator AICAR partially reversed the Dex-induced BAT whitening, attenuated Dex-induced UCP1 downregulation, and improved survival in neonatal rats. The morphology of mitochondria within the BAT of AICAR co-treatment group appeared healthier with abundant and aligned cristae. Unlike CQ, which blocked the clearance of damaged mitochondria at the downstream, AMPK activators clear the damaged mitochondria by improving the autophagy “flux” from both upstream (promoting synthesis of autophagosome) and downstream (activation of mitochondrial fission and thereby improving autophagy). Of note, we found Dex treatment itself already activated the AMPK pathway and autophagosome synthesis; therefore, it implied that the molecular mechanism by which AICAR rescued Dex-induced BAT damage cannot be solely explained by increased autophagy, but rather through activation of abovementioned multiple pathways. However, the damage could only be partially recovered, possibly due to the extensive targets of Dex. We speculated that combined treatment with medication targeting different pathways may potentially improve the therapeutic effects.

Furthermore, because BAT is richly innervated by sympathetic nerve system outflow from the hypothalamus (e.g. under cold exposure), one may argue whether our systemic administration of AMPK activator exerted its effect in the hypothalamus or in the BAT. Literature research showed that the direct effect of AMPK activation in the hypothalamus is to decrease sympathetic outflow to BAT and dampens BAT-mediated thermogenesis 65-71. Therefore, the effect of AMPK activation on thermogenesis is opposite between BAT and hypothalamus. Moreover, AICAR has very low permeability across the blood-brain barrier 72-74. Therefore, the beneficial effect of AMPK activation on thermogenesis in our study is likely mediated through a direct action on the BAT.

A recent study demonstrated the detrimental effects of excessive prenatal glucocorticoid exposure on BAT of offspring in mice 75. Chen *et al.* showed that offspring of mice injected with Dex during the last trimester of gestation had increased DNA methylation in the *Ppargc1a* promotor in neonatal BAT and brown progenitors, resulting in persistently impaired BAT function till 4-month-old. Although their animal model represented prenatal GC exposure (i.e. maternal stress or antenatal steroid), many results were in line with ours. Our experimental model represents the postnatal use of GC in clinical setting for prevention or treatment of bronchopulmonary dysplasia, which lacks effective treatments other than GC and remains a therapeutic challenge to date 76. Whether postnatal Dex therapy is associated with long-term adverse effects on metabolism or thermogenesis warrants further study.

Last but not least, we tried to verify our hypothesis in human infants. We demonstrated that preterm infants exhibited greater diurnal temperature variation during systemic corticosteroid treatment compared with controls. The differences were minimal (median 0.60 vs. 0.50 °C), though, possibly because these infants were nursed in thermo-controlled environments. However, our human study has several limitations. First, the study was a retrospective study. The clinical indications, type of steroid, dose and duration varied. Second, the case number was relatively small, which may be due to the fact that our NICU had relatively low incidence of bronchopulmonary dysplasia (32.8 % among VLBW infants, according to data from 2017-2019). Third, the temperature recordings of our patients were the results after thermo-controlled adjustment by the incubator. Further prospective studies with more direct measurement of BAT function (e.g. infrared camera) are needed. Nevertheless, our human data, at least in part, raise awareness of the possibility of the detrimental effects of postnatal corticosteroids on thermogenesis and temperature regulation in preterm infants. However, further human studies are needed before translation of novel treatments to clinical practices.

## Conclusions

Short course postnatal low dose Dex therapy caused growth retardation and BAT whitening, impaired BAT thermogenesis and led to cold intolerance in neonatal rats. Dex impaired thermogenesis, autophagosome clearance, mitochondrial function and dynamics of BAT in neonatal rats. Our study underscores the therapeutic potential of AMPK activators, which may be due to their multiple effects on mitochondrial fission and autophagy activation. Finally, we found postnatal systemic corticosteroid use to be associated with greater temperature fluctuation in preterm infants, suggesting possible detrimental effects on temperature regulation, which has been overlooked before.

## Supplementary Material

Supplementary figures and tables.

## Abbreviations

AICAR: 5-Aminoimidazole-4-carboxamide ribonucleotide
BAT: brown adipose tissue
BPD: bronchopulmonary dysplasia
BW: body weight
CQ: chloroquine
Dex: dexamethasone
GA: gestational age
GC: glucocorticoid
GR: glucocorticoid receptor
LCFA: long-chain fatty acids
Rapa: rapamycin
UCP1: uncoupling protein-1
WAT: white adipose tissue
