# Supplementary material for: Postnatal Dexamethasone Therapy Impairs Brown Adipose Tissue Thermogenesis and Autophagy Flux in Neonatal Rat Pups
Source: Theranostics. 2022 Jul 25;12(13):5803–19. doi: 10.7150/thno.70752 (PMC9373816; doi:10.7150/thno.70752)
Supplement: Supplementary file 1 — Supplementary figures and tables. [file thnov12p5803s1.pdf]

Figure S1

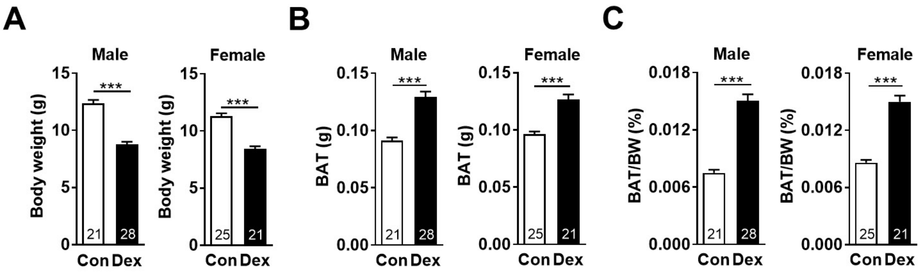

Figure S1. Effect of Dex on BAT in male and female neonatal rats. (A) Body weight. (B) BAT weight. (C) BAT/body weight ratio. Numbers of rats in each group were indicated within bars. Data were expressed as mean ± SEM with statistics analyzed by student's *t*-test (****p* < 0.001).

Figure S2

A

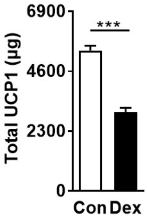

Figure S2. Effect of Dex on total UCP1 protein. Total UCP1 protein levels was obtained from the data of quantified immunoblots (that yield the level of UCP1 protein per mg tissue protein) multiplied by the total protein content in the tissue. Con, n = 3; Dex, n = 3. Data were expressed as mean ± SEM with statistics analyzed by student's *t*-test (****p* < 0.001).

Figure S3

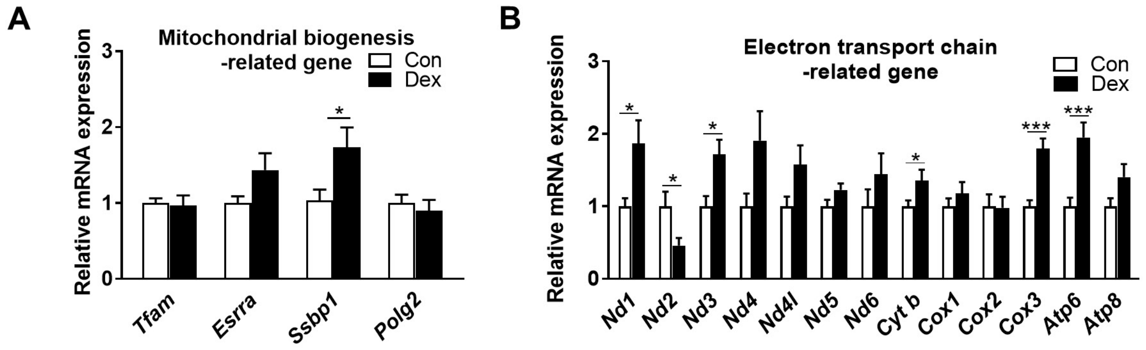

Figure S3. Effect of Dex on BAT mitochondrial biogenesis and electron transport chain. Expression of (A) mitochondrial biogenesis-related genes (n = 12) and (B) mitochondrial electron transport chain components (n = 10). mRNA levels were expressed relative to average expression in the control rats. Data were expressed as mean ± SEM with statistics analyzed by student's *t*-test (**p* < 0.05 and ****p* < 0.001).

Figure S4

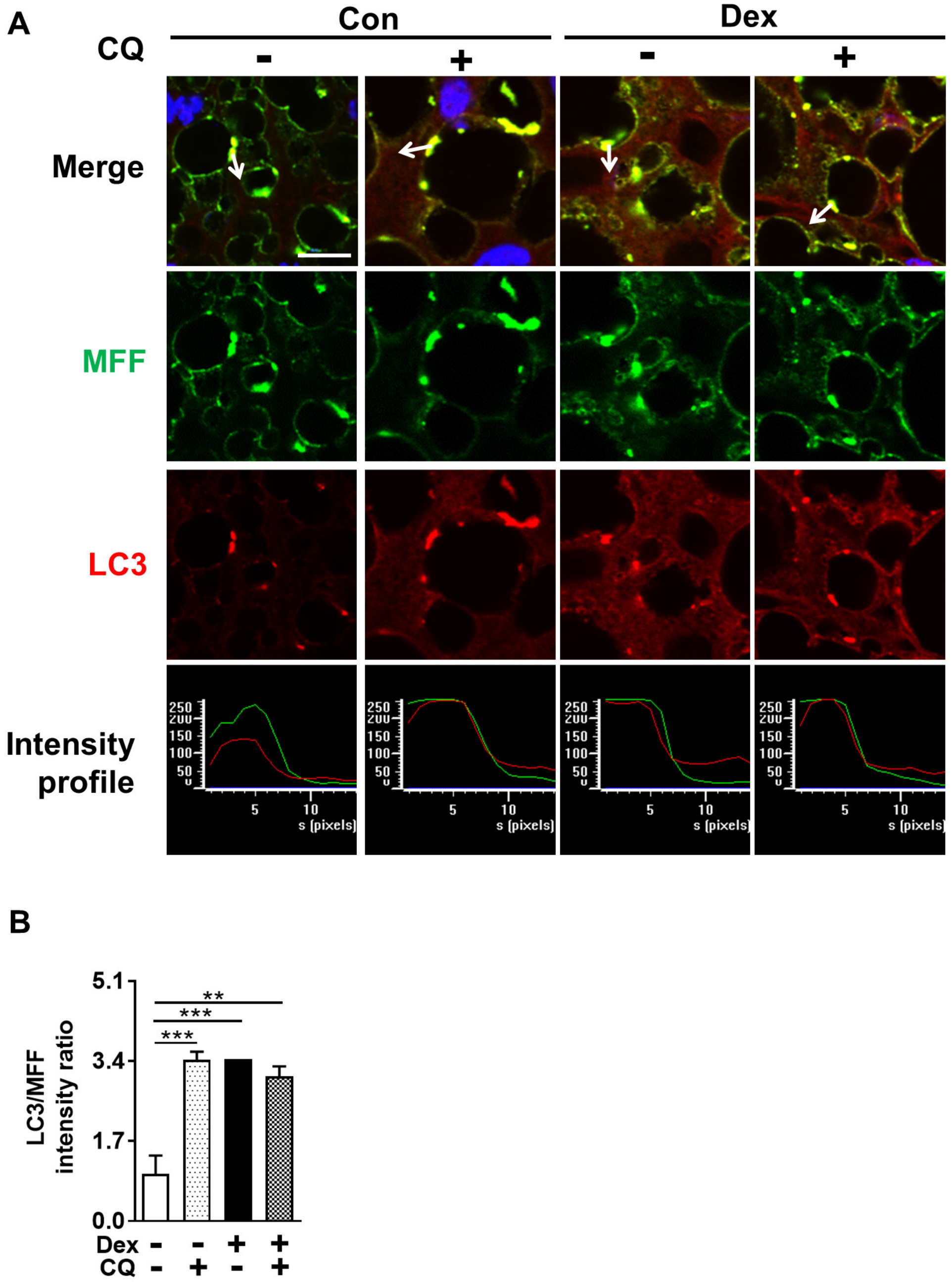

Figure S4. Effect of Dex on BAT mitophagy. (A) Immunofluorescence image of colocalization of mitochondrial fission factor (MFF) (mitochondrial marker) and LC3. Scale bar: 5 μm. Intensity profile was calculated by ImageJ software. (B) Quantitative analysis. Data were expressed as mean ± SEM with statistics analyzed by one-way ANOVA (***$p < 0.001$).

Figure S5

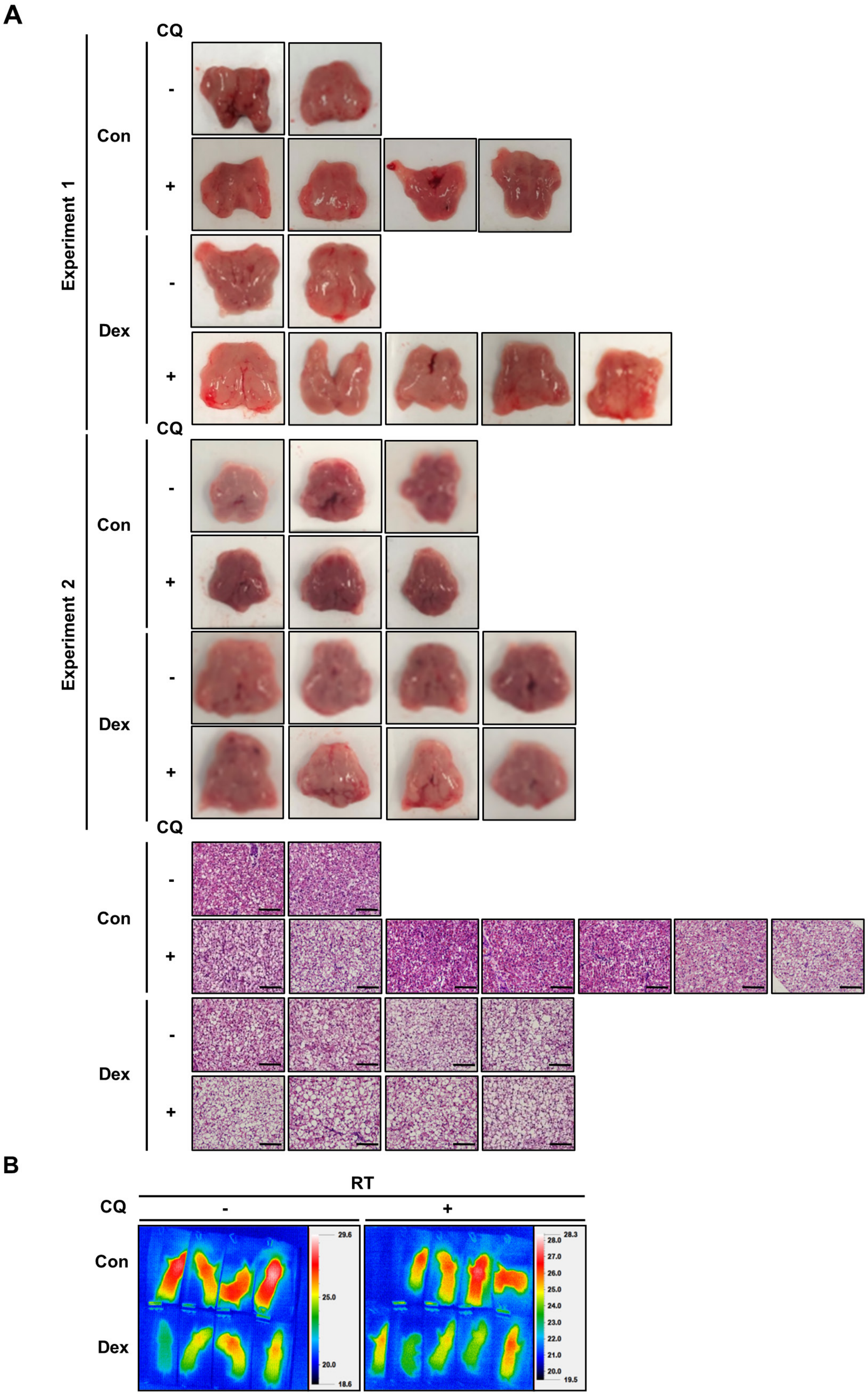

Figure S5. Effect of Dex on BAT autophagy and co-treatment with CQ. (A) Original pictures with gross morphology in upper panels and H&E stain in lower panels in two sets of CQ co-treatment experiments. (B) Evaluation of body temperature with CQ by infrared thermo-imaging before the beginning of cold challenge (RT). Con, n = 4; Con + CQ, n = 4; Dex, n = 4; Dex + CQ, n = 5.

Figure S6

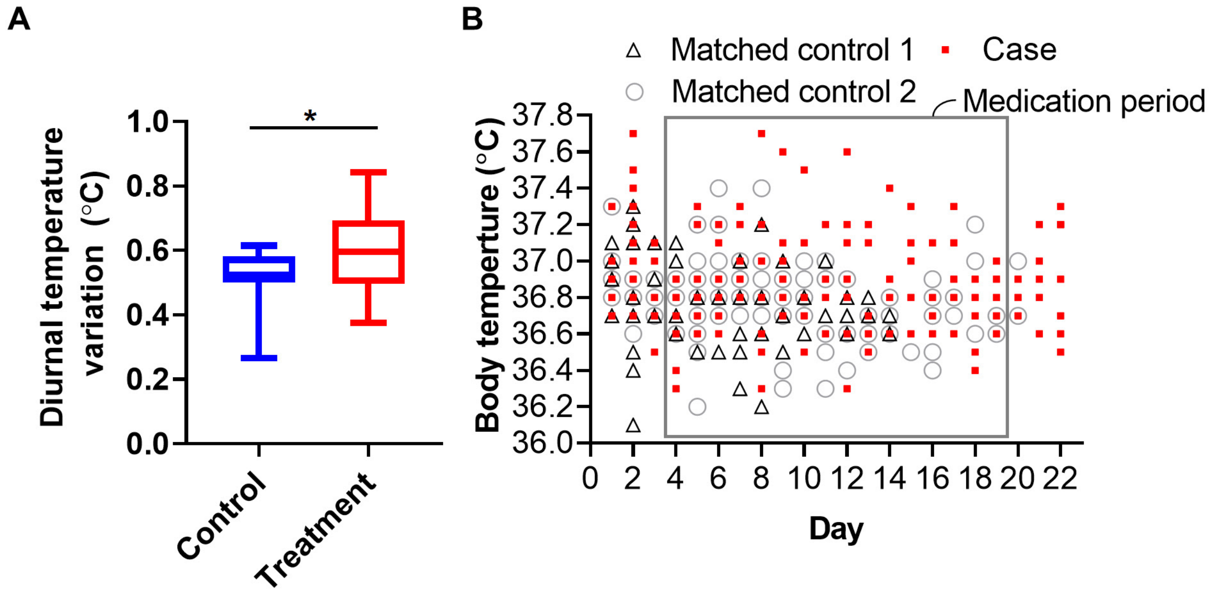

Figure S6. Daily temperature fluctuation during systemic corticosteroid treatment (treatment group) or treatment-naïve controls (control group) at equivalent postnatal age. (A) Diurnal temperature variation. Expressed as box-plot showing median, 25th and 75th percentile, min and max values and calculated by Mann-Whitney U test (*$p < 0.05$). (B) Example of raw temperature records starting 3 days before corticosteroid treatment (or equivalent postnatal age in control) until 3 days after treatment ended in one case and two gestational age- and sex-matched controls. Temperature records during corticosteroid treatment were indicated as "medication period" and expressed as the scatter plot of each temperature record per day. Temperature records were incomplete after the medication period in controls because the patients were discharged from the hospital.

Table S1. Rat primer sequences designed for PCR.

Genes	Forward	Reverse
Ucp1	CATCATCAACTGTACAGAGC	ATCTCGTTTTTACCACATCC
Prdm16	ATCTACAGCAGGGTAGAAAAGCG	TCTCCGTCATGGTTTCTATG
Cidea	GAACTTATCAGCAAGACTCTG	ATCATGAAGTGTGTGTTGTC
Adrb3	CAACAGGTTTGATGGCTATG	CACTCTGAGCAGAAATCAAG
Atf2	CCTTCTGTTGTAGAAACAACTC	CACATTGGGAACCTGTAATG
Cebpa	AAGAGCCGAGATAAAGCC	GTCATTGTCACTGGTCAAC
Cebpb	AATCACTTAAAGATGTTCCTGC	AAAATGTCTTCACTTTAATGCTC
Cebpd	AATGGTAGCGTTTTCTACG	AAAGTCTGTCGGAAAAGTC
Ppargc1a	AGGTATGACAGCTATGAAGC	GTGTCAGGTCTGATTTTACC
Fasn	TATGCTTCTTCGTGCAGCAGTT	GCTGCCACACGCTCCTCTAG
Acaca/ACC	GCCTCTTCCTGACAAACGAG	GGACTGCCGAAACATCTCTG
Mlxipl/Chrebp	CGGGACATGTTTGATGACTATGTC	CATCCCATTGAAGGATTCAAATAAA
Nr1h3/LXRa	AGGAGTGTCGACTTCGCAAA	CTCTTCTTGCCGCTTCAGTTT
Srebf1	CTTCCCAGCCCCTCAGATA	TGTGACTGGCTCACCGTAGA
Leptin	TACTGCTGCGTCTGAAAATCCA	TGAAGCCCAGGAATGAAGTC
Fabp4	CACCGAGATTTCCTTCAAACT	GCCATCTAGGGTTATGATGC
Elovl6	ACAATGGACCTGTCAGCAAA	GTACCAGTGCAGGAAGATCAGT
Acsl1	TCTTCCCTGTGGTTCCCAG	AAGCTCCGCCTCTTTCCTTT
Cpt1a	TGCACTACGGAGTCCTGCAA	GGACAACCTCCATGGCTCAG
Cpt1b	TATTAAGAACACGAGCCAAC	GTAGCAAGTCTGTCTCTTTG
Cpt2	GACCAAAGAAGCAGCGATGG	GCAGCCTATCCAGTCATCGTG
Acadl/LCAD	ATGGCAAAATACTGGGCATCTG	TGTCTTGCGATCAGCTCTTTCA
Acadm/MCAD	GAGTGCCTAAGGAAAATGTG	AGAAATGAAACTCCTTGGTG
Ppara	ATGCCTTAGAACTGGATGACA	GCAACTTCTCAATGTAGCCTA
Tfam	GCTTGGAAAACCAAAAAGAC	CCCAAGACTTCATTTCATTGT
Esrra	ATGAGTGTGAGATCACCAAGCG	CCGCCGCTTGTACTTCTGTC
Ssbp1	CAAAAGACAACATGGCACAGAATATC	TAGTCCACTTTGCCTTCCACAAAT
Polg2	ACAGTGCCTTCAGGTTAGTTTCTCC	ACACTCCAATCTGAGCAAGGC
Nd1	CACCCCCTTATCAACCTCAA	ATTTGTTTCTGCGAGGGTTG
Nd2	AACCCAAGCTACAGCCTCAA	GAAATTGCGAGAATGGTGGT
Nd3	TAACATCACCTTATCCTTTATCCTC	GGCAGTTGCTATTATTGTAGTGG
Nd4	TCCCACTCTTAATTGCCCTC	GAGGATGATGAATGGGTAGG
Nd4l	CTCCAACTCCATAATCTCCATAAC	GGCTAAACCTACTGCTGCTTC
Nd5	ATCGAAGCCATCAACACGTG	GCAGTTATGGATGTGGCGATT
Nd6	AGCCTTCACCTATTTATG	CACCCAGCCACCACTATC
Cytb	GGAGTTCCAAGATGCCTGGA	CCACTAACATCACCACCTCATAGC
mt-Cox1	TCACAGTAGGGGGCCTAACA	GGCTTTTGCTCATGTGTCATT
mt-Cox2	GCTTACAAGACGCCACATCACC	CGTAGGGAGGGAAGGGCAAT
mt-Cox3	TCTTCTTTGCCGGATTTTTC	ATGGTTTCGGTTGCCTTCTA
mt-Atp6	CGAACCTGAGCCCTAATA	GTAGCTCCTCCGATTAGA
mt-Atp8	TGCCACAACTAGACACATCCA	TGTGGGGGTAATGAAAGAGG

Table S2. List of antibodies used in experiments.

Antigen	Host	Cat.	Source
UCP1	Rabbit	ab209483	Abcam
PPARγ	Rabbit	#2443	Cell Signaling
PGC-1	Mouse	AB3242	Millipore
TRα/β	Rabbit	sc-772	Santa Cruz
DIO2	Rabbit	ab77779	Abcam
Phospho-p38 MAPK (Thr180/Tyr182)	Rabbit	#9211	Cell Signaling
p38 MAPK	Rabbit	#9212	Cell Signaling
Phospho-ATF-2 (Thr71)	Rabbit	#9221	Cell Signaling
ATF-2	Rabbit	GTX11908	GeneTex
Phospho-CREB (Ser133)	Rabbit	#9191	Cell Signaling
CREB	Rabbit	sc-186	Santa Cruz
Phospho-HSL (Ser565)	Rabbit	#4137	Cell Signaling
Phospho-HSL (Ser660)	Rabbit	#4126	Cell Signaling
HSL	Rabbit	#4107	Cell Signaling
PLA2	Rabbit	GTX103717	GeneTex
Mitofusin 1 (MFN1)	Mouse	Ab57602	Abcam
Mitofusin 2 (MFN2)	Rabbit	M6444	Sigma
OPA1	Mouse	612607	BD
Phospho-DRP1 (Ser616)	Rabbit	#3455	Cell Signaling
Phospho-DRP1 (Ser637)	Rabbit	#4867	Cell Signaling
DLP1(DRP1)	Mouse	611113	BD
FIS1	Rabbit	AXL-201-1037	ENZO
MFF	Goat	sc-168593	Santa Cruz
Phospho-MFF (Ser146)	Rabbit	#49281	Cell Signaling
MID49 (SMCR7)	Mouse	sc-515759	Santa Cruz
MID51 (SMCR7L)	Mouse	sc-514135	Santa Cruz
Porin	Rabbit	PC548	Merck
ATG5	Rabbit	GTX113309	GeneTex
ATG7	Rabbit	GTX32459	GeneTex
BECN1	Rabbit	#3738	Cell Signaling
LC3B	Rabbit	#3868	Cell Signaling
p62	Rabbit	PM045	MBL
α-tubulin	Mouse	SI-T5168	Sigma
β-actin	Mouse	A5441	Sigma
GAPDH	Mouse	MAB374	Millipore
